# Effects of six-week stroboscopic training program on visuomotor reaction speed in goal-directed movements in young volleyball players: a study focusing on agility performance

**DOI:** 10.1186/s13102-024-00848-y

**Published:** 2024-02-29

**Authors:** Michał Zwierko, Wojciech Jedziniak, Marek Popowczak, Andrzej Rokita

**Affiliations:** 1 Department of Team Sports Games, Wroclaw University of Health and Sport Sciences, Wroclaw, Poland; 2 Institute of Physical Culture Sciences, University of Szczecin, Szczecin, Poland

**Keywords:** Reaction time, Visual training, Team sports, Agility, Stroboscopic eyewear

## Abstract

**Background:**

In team sports, deficits in visuomotor reaction speed are considered a significant and modifiable risk factor that can lead to decreased performance and an increased risk of injuries. Thus, identifying effective methods to enhance visuomotor abilities is crucial. The main objective of this research was to investigate the impact of a six-week stroboscopic intervention on visuomotor reaction speed in goal-directed specific movements based on agility among young volleyball players. Additionally, the study aimed to explore the impact of saccade dynamics on visuomotor reaction speed performance throughout the experiment.

**Methods:**

There were 50 athletes (26 males and 24 females) with an average age of 16.5 years (± 0.6) who participated in this study. Over a six-week training period, athletes performed volleyball-specific training either wearing stroboscopic glasses (intervention) or under normal visual conditions (control). Prior to and after the training period, the agility tests based on change-of-direction speed (CODS) and reactive agility (RA) were used to identify visuomotor reaction speed performance. To measure agility performance a five-repetition shuttle run to gates was conducted. The REAC-INDEX, which represents visuomotor reaction speed, was analyzed as the resulting difference between the CODS test and the RA test. To elicit saccadic dynamics, a laboratory visual search task was performed.

**Results:**

A significant GROUP×TIME interaction was observed for the REAC-INDEX (*p* = 0.012, ηp^2^ = 0.13). ANCOVA analyses revealed significant GROUP differences, indicating improved post-training REAC-INDEX results (*p* = 0.004, *d* = 0.87), regardless of gender. Training-induced modulations in saccade acceleration did not reach significance, but a significant relationship was observed between changes in saccade acceleration and changes in the REAC-INDEX (*r* = -0.281, *p* = 0.048), indicating that higher performance gains following training were associated with a stronger increase in saccade acceleration.

**Conclusions:**

This study demonstrates that stroboscopic training effectively enhances visuomotor reaction speed in goal-directed movements based on agility. Furthermore, visuomotor reaction speed gains could potentially be mediated by saccade dynamics. These findings provide valuable insights into the effectiveness of stroboscopic eyewear for training sport-specific visuomotor skills among young volleyball players.

**Supplementary Information:**

The online version contains supplementary material available at 10.1186/s13102-024-00848-y.

## Background

In sports, athletes need to react to visual information related to motion. The ability to rapidly process visual input into motor responses is particularly crucial in ball team sports, where perceiving and analyzing motion attributes such as ball speed, trajectory, and spin are vital for achieving success, especially under fatigue conditions [1]. Furthermore, due to the dynamic nature of the game, athletes need to swiftly identify objects and effectively scan the visual field for relevant points of interest to make optimal motor decisions [2]. Studies have consistently shown that athletes exhibit superior visuomotor reaction performance compared to non-athletes or novices in multiple sports, including soccer [3], baseball [4], volleyball [5, 6], basketball [7] and handball [8, 9]. Importantly, deficits in visuomotor reaction performance are recognized as a significant and modifiable injury risk factor [10, 11]. This becomes particularly crucial during movements involving deceleration, cutting, or landing, which frequently occur in situations of high uncertainty, such as defending [12]. Therefore, the search for new methods of training visuomotor abilities is important from the perspective of both athletic performance and the athletes’ health.

Visuomotor reaction speed refers to the ability to perform rapid and precise movements of the body or a part of the body in response to a visual stimulus. It encompasses both the reaction time—from the onset of the stimulus to the initiation of movement—and the time required for the execution of the movement [13]. This ability is crucial in team sports, as it assists players in executing goal-directed movements. These movements involve mechanisms where visual input triggers motor actions [14], facilitating coordinated movements to achieve specific targets during the game. This integration of vision and action is essential for players to respond effectively to the dynamic environment of team sports. The effectiveness of responses to visual stimuli during sport-specific actions in the game typically relies on agility-based maneuvers [15]. Agility in sports encompasses rapid, full-body movements involving alterations in velocity and direction as a response to stimuli [16]. According to this definition, two key aspects of agility can be identified: the change-of-direction speed (CODS) and reactive agility (RA). CODS is typically pattern-based, pre-planned, and closed skill [17]. The assessment of CODS is predominantly influenced by an individual’s physical attributes [18]. In contrast, RA focuses specifically on the ability to react and change direction in response to external cues or stimuli, such as an opponent’s movement or a ball trajectory [19]. RA pertains to spontaneous movements that are influenced by factors such as motor abilities, running technique, and perceptual-cognitive traits [20–22]. In the context of agility-related aspects, the key differentiation between RA and CODS lies in the time required for visuomotor reaction processes to occur when responding to an unforeseen signal. This distinction has been quantified in research as the REAC-INDEX, which reflects the difference in the time taken to complete RA and CODS tasks while assuming an identical movement structure for both tasks [23, 24]. In this study, the changes in these aspects of agility resulting from stroboscopic training intervention will be examined.

Stroboscopic training, a method that imposes increased demands on the visuomotor system through intermittent visual stimulation during motor tasks, has the potential to lead to improved performance under normal visual conditions [25]. This technique utilizes stroboscopic glasses, also known as shutter glasses, which purposefully reduce visual information by alternating between transparent and opaque states. Athletes can integrate these glasses into their sport-specific tasks, thereby facilitating the practice and enhancement of their visual, perceptual-cognitive, and motor processing in real-world scenarios. The fundamental principle of stroboscopic training involves delivering a progressive stimulus to the visual system by adjusting the frequency and duty cycle of the stroboscopic goggles. This approach deliberately increases the challenge of performing motor exercises, aiming to elicit adaptive responses [26]. Such adaptations are thought to optimize visuomotor processing efficiency in standard visual conditions, ultimately leading to improved athletic performance. Recent research suggests that stroboscopic training has a significant impact on both perceptual-cognitive and motor factors [26–29], including enhancing postural control in dynamic balance tasks [30]. Current reports on the impact of stroboscopic training on athletes’ sport-specific movements (far transfer) are still limited and do not provide clear findings. For instance, an experimental study demonstrated that stroboscopic training resulted in improved on-field performance in badminton [26]. However, the effects of the training were not significantly different from those observed in the control group. On the other hand, the study in ice hockey, which included on-ice field tests such as shooting accuracy, reaction time, and puck handling, demonstrated that stroboscopic training resulted in an average 18% improvement in on-ice skill performance for the intervention group (*n* = 6), while the control group (*n* = 5) showed no improvement [31]. However, this study had several limitations, especially regarding the small number of participants (*n* = 11), as well as the lack of uniformity in the stroboscopic training procedure. In this case, participants were free to use the stroboscopic eyewear as they wished, provided they wore the eyewear for at least 10 min each day. Furthermore, stroboscopic vision may enhance training effects in football-specific skills, especially among highly skilled players, as no significant improvements were observed among less-skilled players [32]. However, due to the limited number of studies confirming the effectiveness of stroboscopic training on athletes’ on-field specific skills, it is crucial to conduct further research in this area to explore its potential applications in broader sports training contexts.

The current study aims to investigate the impact of a six-week stroboscopic intervention on visuomotor reaction speed in specific agility field tasks in young volleyball players. Our objective is to investigate the assessment of changes in visuomotor reaction speed, specifically measured using the REAC-INDEX, following a stroboscopic training program. Additionaly, the present study aims to explore the potential relationship between alterations in oculomotor function, specifically measured saccade dynamics, and changes in post-training REAC-INDEX results. Saccadic eye movements are key in directing high acuity foveal vision to significant regions of visual space and are the primary means of conducting visual searches. They facilitate rapid shifts of fixation from one target to another, directing gaze toward objects of interest and aiding in the swift acquisition of visual information [33]. This process, in which saccade dynamics plays an integral role, is crucial for efficient visual scanning and quick attentional shifts in response to visual stimuli. Such capabilities are vital in various real-world scenarios, including fast-paced sports like volleyball, where adapting quickly to changing visual objects and effectively searching the visual field are essential for performance [34]. In our research, the saccade acceleration parameter was analyzed to describe saccade dynamics. Building upon previous findings [26–28], we hypothesized that training with the use of stroboscopic eyewear would be more effective in improving visuomotor reaction speed in on-field task in relation to the same training without stroboscopic eyewear.

## Methods

The research methodology was largely based on a previous study conducted by the authors [35]. In that study, we provided an extensive description of the studied groups, measurement methods, and procedural details. Here, we present a concise summary of those components.

### Participants

The study involved 50 volleyball players, including 26 males and 24 females, with an average age of 16.5 years old (± 0.6 years standard deviation). The participants were randomly assigned to either a stroboscopic or non-stroboscopic group, with an almost equal number of males (*n* = 13) and females (*n* = 12) ensured in each group. The stroboscopic group participants had average heights of 180.2 cm (± 8.2 cm) and weights of 74.3 kg (± 10.4 kg). The non-stroboscopic group showed similar physical stats, with an average height of 181.9 cm (± 8.1 cm) and weight of 71.6 kg (± 8.9 kg). Both groups had comparable experience in volleyball training, with an average of 6.7 years (± 1.1) for the stroboscopic group and 6.6 years (± 1.3) for the non-stroboscopic group. All participants trained in volleyball on a regular basis, at least 5 days a week, and participated in official volleyball federation competitions during the season.

### Agility performance (field-test)

Performance in CODS and RA was measured using a five-time shuttle run to gates, employing the Fusion Sport Smart Speed System (Fusion Sport, Coopers Plains, QLD, Australia). The layout of this test was designed based on methodologies detailed in prior studies [35, 36].

**Fig. 1:**
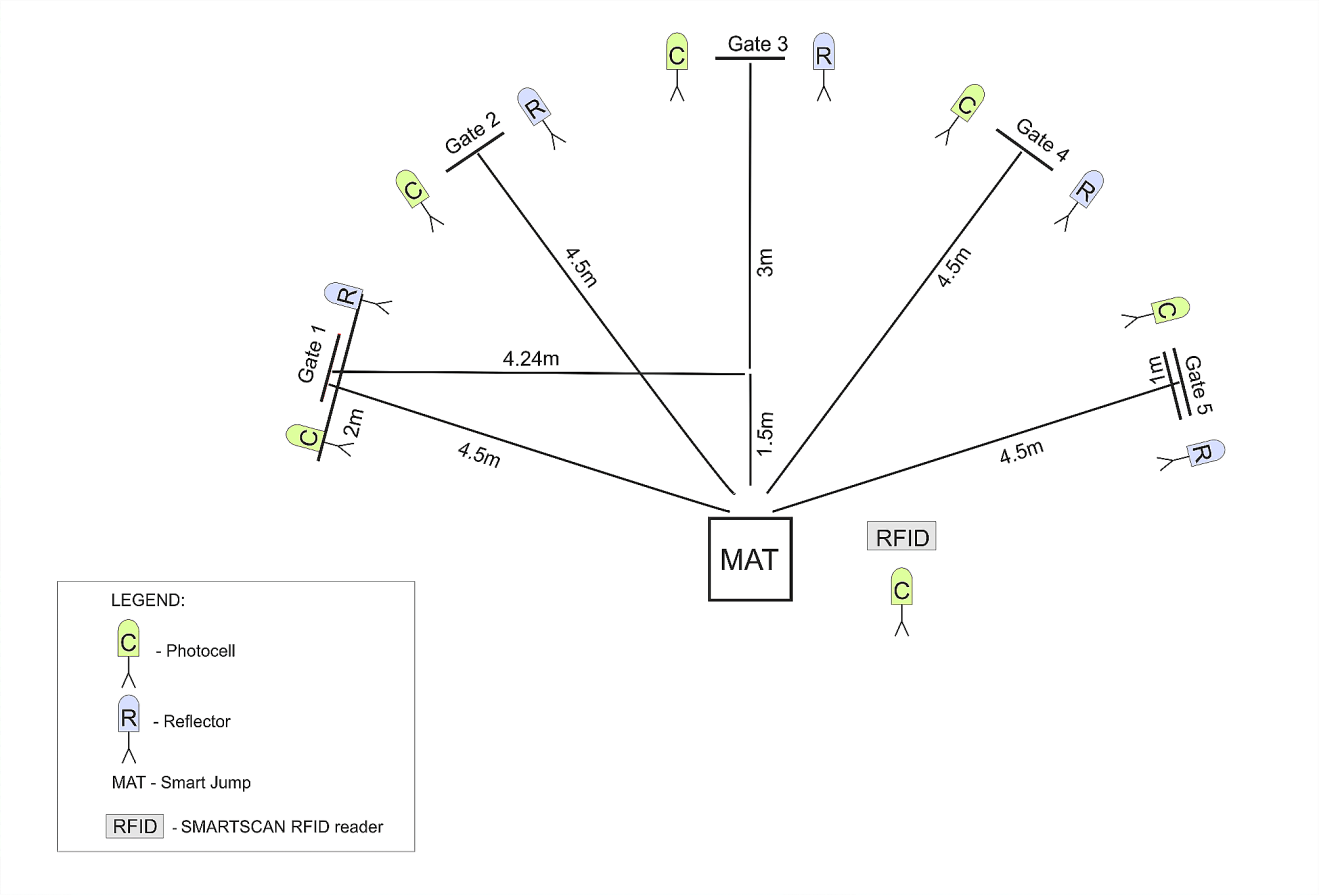
A schematic of the agility tests. The system includes an RFID reader for athlete tag recognition, electronic gates equipped with a photocell (C), an infrared transmitter and light reflector (R), and a smart jump mat (MAT) integrated with a photocell. The software records movement times during the runs. Participants perform five runs to designated gates, each time starting and returning to the smart jump mat, following a preplanned route for the CODS with gate order 1-5-2-4-3 and a random sequence for the RA

In the first scenario (CODS performance), the test was preplanned, and the participant ran to the gates in a specific order (1-5-2-4-3). In the second scenario (RA performance), the participant ran to randomly selected gates, and the order varied for each participant. Both the CODs test and the RA test were repeated twice, and the best result from each trial was used for analysis. The order of performing the RA and CODS tests was randomized for all participants. Figure 1 provides a visual representation of the schematic for the agility tests.

To determine the visuomotor reaction speed in field test, the REAC-INDEX was calculated. The REAC-INDEX represents the time difference between the RA test result and the CODS test result, as previously described [23]. The REAC-INDEX was calculated using the following formula: REAC-INDEX [s] = RA [s] - CODS [s].

### Saccade dynamics (lab-test)

Saccade dynamics were evaluated during a visual search task performed in a free-viewing paradigm. The task involved identifying a red letter ‘E’ from a field of 47 distractors displayed on a screen. Saccadic eye movements were recorded and analyzed, as described previously [37]. The saccade dynamics were evaluated by calculating the average saccade acceleration (deg/s^2^). Binocular gaze data were collected at a rate of 60 Hz (SMI ETG 2w, Germany) using a portable eye-tracking system. A standard three-point calibration procedure was conducted binocularly.

### Procedure

The study involved a six-week training period, with training sessions conducted three times per week. Both the stroboscopic and non-stroboscopic groups performed identical sport-specific exercises. However, they experienced different visual conditions. The experimental (stroboscopic) group wore stroboscopic glasses during the exercises, while the control (non-stroboscopic) group completed the same exercises without wearing any glasses. Three volleyball-specific training protocols with ball exercises were conducted. Protocol 1, “wall passing drills,” included tasks involving reaction time exercises such as visual search. Protocol 2, “partner passing drills,” featured tasks in a frontal position, incorporating reaction time exercises with external light stimuli, tennis balls, and time pressure. Protocol 3, “passing rotation drill,” involved two passing forms (overhead and forearm passes) with directional changes under forced time pressure. The protocols had durations of 25 and 30 min, with 2.5-minute intervals and 2.5-minute breaks. The stroboscopic group used stroboscopic eyewear (Senaptec Strobe, Beaverton, USA) during the exercises. The glasses were modulated for frequency (Hz) and duty cycle (%) to prevent adaptation effects. Task difficulty increased gradually over six weeks (week 1: 15 Hz, 50%; week 2: 13 Hz, 50%; week 3: 11 Hz, 50%; week 4: 10 Hz, 50%; week 5: 9 Hz, 60%; week 6: 9 Hz, 70%). Further details of the research procedure were described in our previous study [35].

**Fig. 2:**
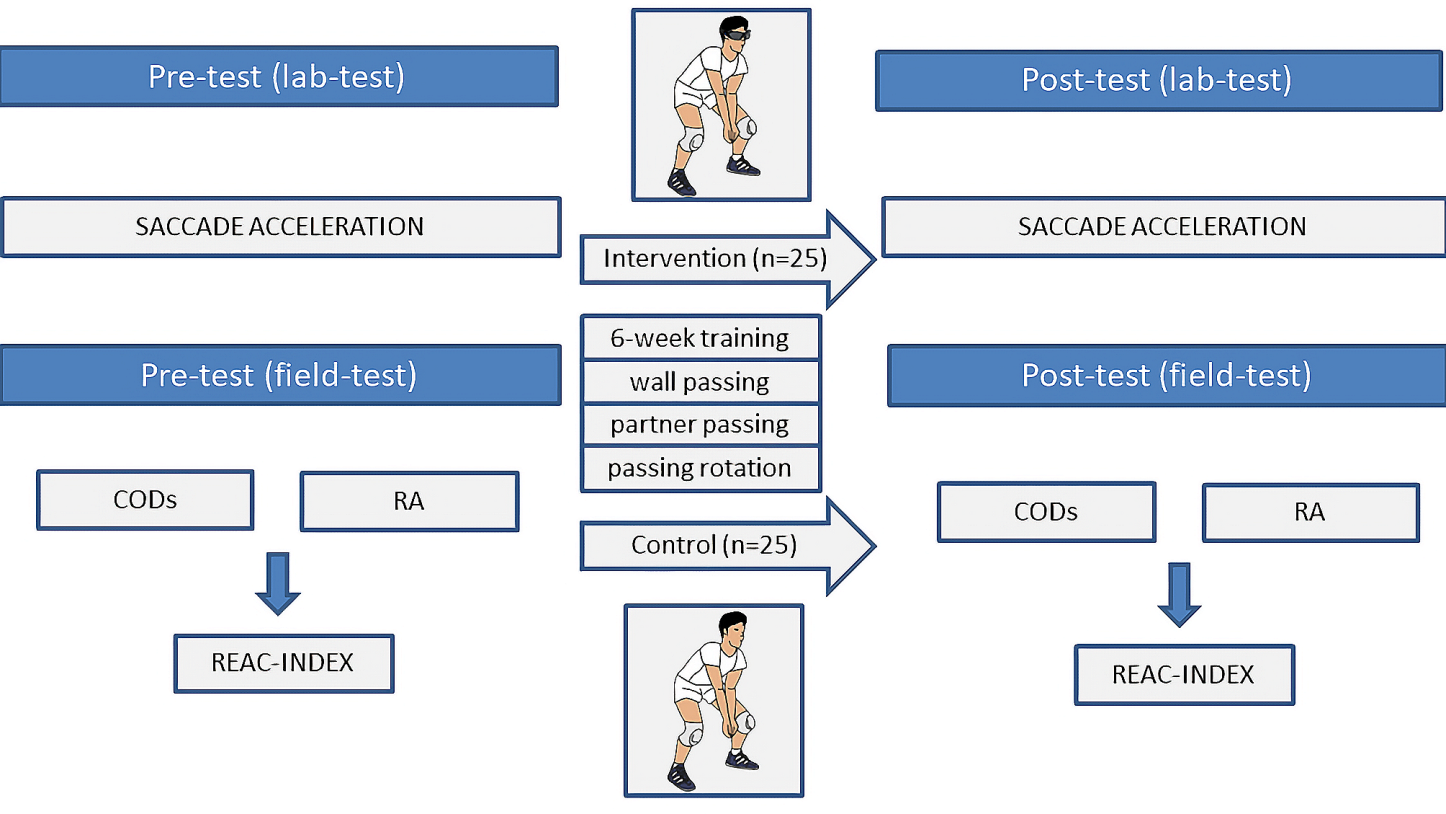
Overview of the experimental protocol for the study. After undergoing a pre-test, athletes were randomly assigned to either the intervention (stroboscopic) group (*n* = 25) or the control (non- stroboscopic) group (*n* = 25) within their respective gender groups Note: CODS– change-of-direction speed, RA– reactive agility, REAC-INDEX– visuomotor reaction speed

Pre- and post-tests were conducted, which included a laboratory test for saccadic acceleration, field tests for agility performance (CODS, RA), and calculation of the REAC-INDEX as a measure of visuomotor reaction speed. Figure 2 provides an overview of the experimental protocol.

### Statistical methods

Descriptive statistics were used to present means and standard deviations. Normality of data was tested using the Shapiro-Wilk test, and homogeneity of variances was verified using the Levene test (*p* > 0.05). To investigate the impact of stroboscopic training on sport-specific visuomotor performance, ANOVAs were conducted with the between-subjects factor GROUP and the within-subjects factor TIME for CODS, RA, REAC-INDEX, and saccade acceleration. Additional analyses of covariance (ANCOVA) were performed for CODS, RA, REAC-INDEX, and saccade acceleration. Post-training performance was used as the dependent variable, while group, gender, and pre-training performance were considered as categorical factors and covariate, respectively. The Holm-Bonferroni procedure was applied for post-hoc comparisons, with a significance level set at *p* < 0.05. Effect sizes were reported using Cohen’s d for t-tests and partial eta squared (ηp²) for F-tests. Cohen’s criteria were used to interpret effect sizes, with values of 0.2, 0.5, and 0.8 representing small, medium, and large effects, respectively, for Cohen’s *d*. For partial eta squared, values of 0.01, 0.06, and 0.14 represented small, medium, and large effects, respectively. Pearson product-moment correlation analysis was used to determine the relationship between training-induced changes in saccade dynamics and visuomotor adaptation. All statistical analyses were performed using JASP software (version 16.1).

## Results

The ANOVA results examining agility parameters revealed that there was no statistically significant main effect of TIME on CODS performance (F_1,48_ = 2.07, *p* = 0.156, ηp^2^ = 0.04). However, notable main effects were observed for RA results (F_1,48_ = 12.64, *p* < 0.001, ηp^2^ = 0.21) and REAC-INDEX (F_1,48_ = 4.26, *p* = 0.044, ηp^2^ = 0.08). Regarding the main effects of GROUP, only RA results reached the significance level (F_1,48_ = 4.34, *p* = 0.043, ηp^2^ = 0.08). Additionally, significant GROUP × TIME interactions were observed specifically for REAC-INDEX (F_1,48_ = 6.87, *p* = 0.012, ηp^2^ = 0.13).

**Fig. 3:**
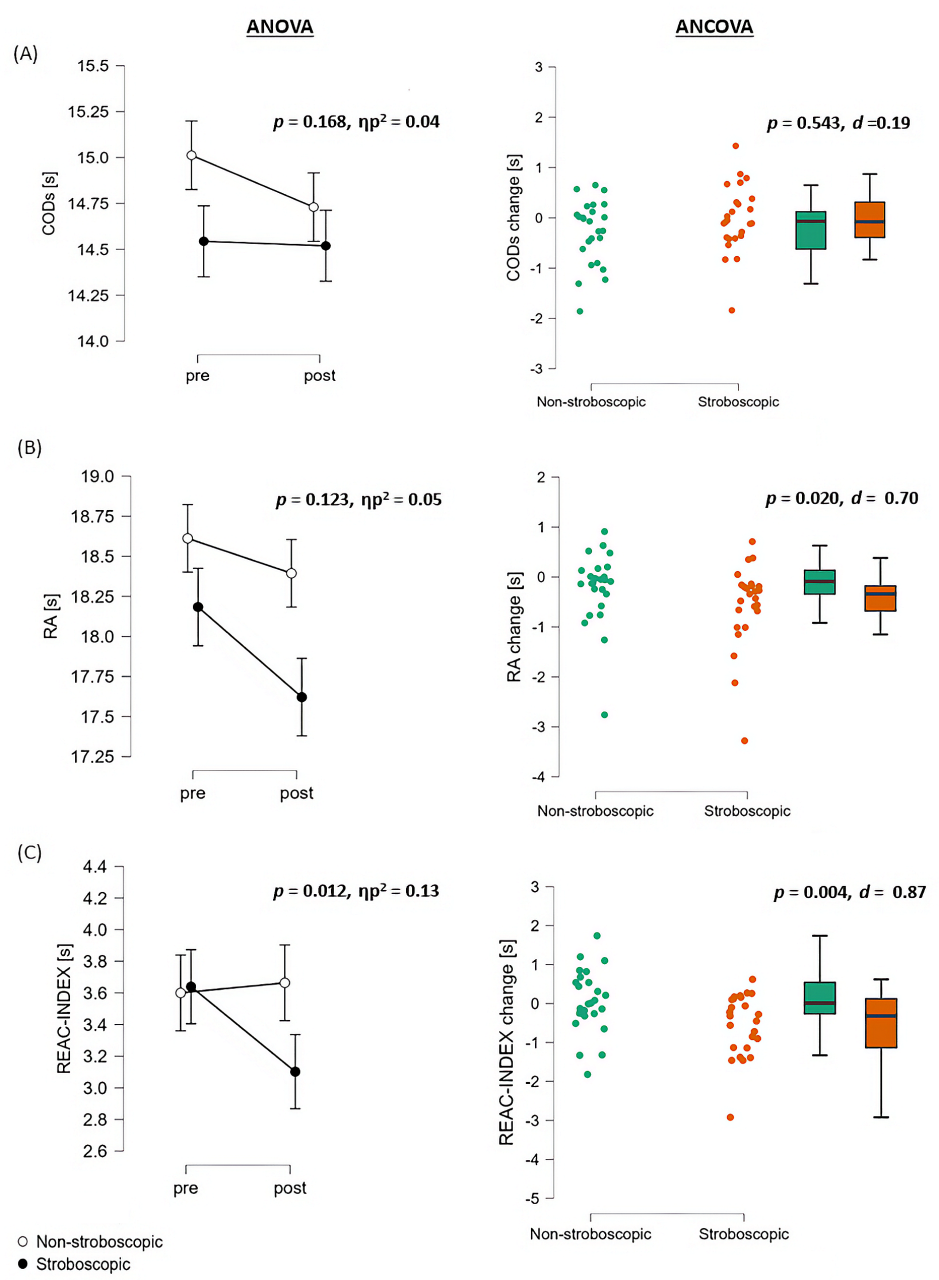
Results obtained from the ANOVA, ANCOVA, and raincloud plots of individual data showing single-subject training-induced changes in **(A)** change-of-direction speed (CODS), **(B)** reactive agility (RA), and **(C)** REAC-INDEX for intervention (stroboscopic) and control (non-stroboscopic) groups. In the right column, boxes represent the interquartile range, and whiskers indicate the overall range of values in each group; the vertical line in each box represents the median

ANCOVA analyses revealed significant GROUP differences, as reflected by improved post-training RA results (F_1,45_ = 5.82, *p* = 0.020, ηp^2^ = 0.12) as well as REAC-INDEX (F_1,45_ = 9.35, *p* = 0.004, ηp^2^ = 0.17) in the stroboscopic group. When considering the pre-training performance in terms of CODS, RA, and REAC-INDEX as a covariate, there were no statistically significant differences observed between genders. Gender was not a factor that interacted with the effects of GROUP. The ANOVA and ANCOVA results are presented in Fig. 3.

The results from the visual search lab-test, designed to initiate saccadic movements, indicate no significant changes (*p* < 0.05) before and after the intervention across the studied groups. The mean reaction times for trials where the target was present (the red letter ‘E’) in the saccadic group were 5.180 ± 0.683s and 4.795 ± 0.924s for pre- and post-tests, respectively. In contrast, for the non-stroboscopic group, these times were 5.068 ± 0.978s before the intervention and 4.999 ± 0.706s afterwards. For trials where the target was absent, the mean reaction times in the saccadic group were 7.423 ± 0.689s before the intervention and 7.147 ± 0.772s after it. Meanwhile, in the non-stroboscopic group, the reaction times were 7.296 ± 0.827 before the intervention and 7.163 ± 0.831s afterwards.

**Fig. 4:**
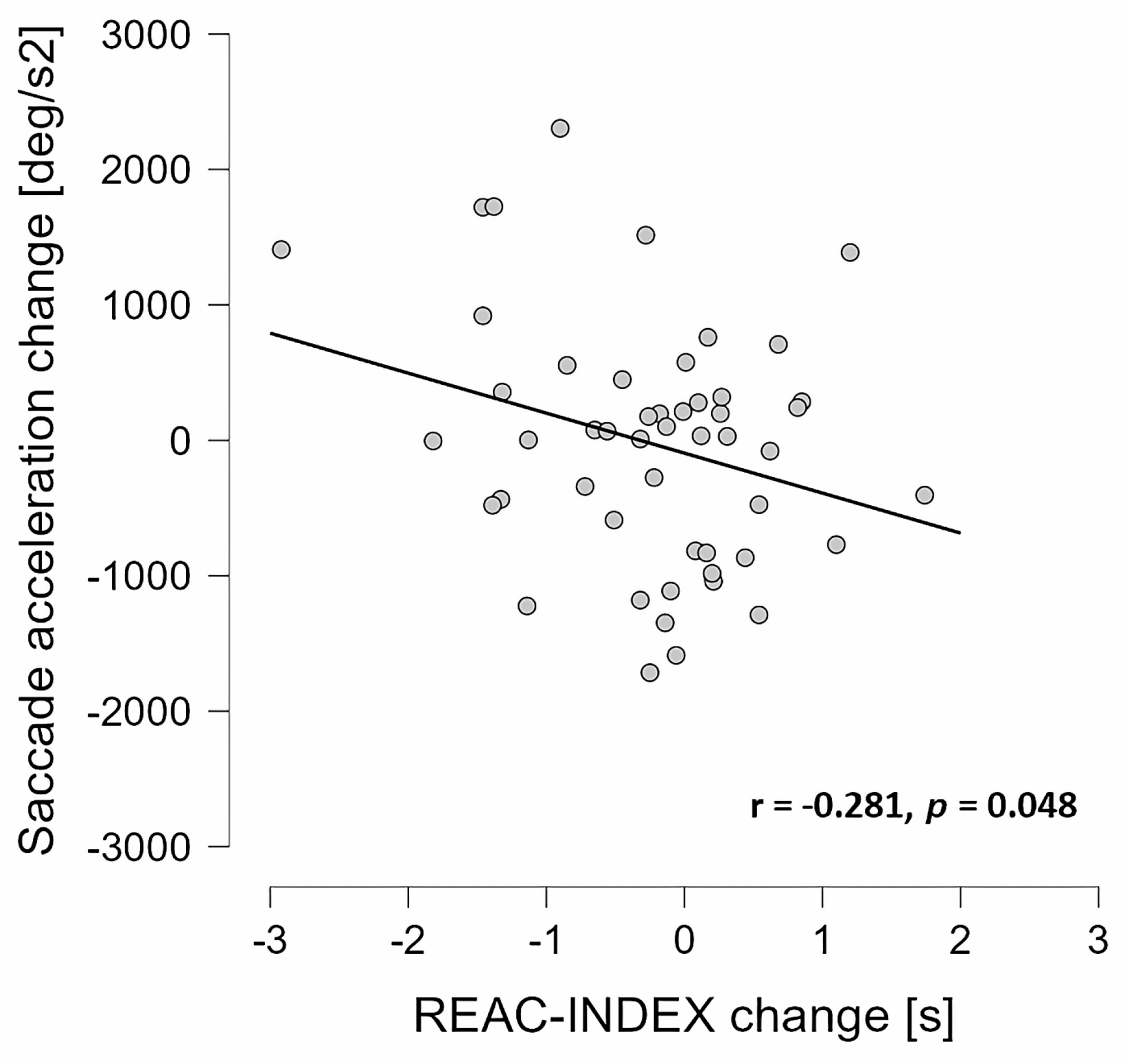
Correlation between changes in saccade acceleration and changes in REAC-INDEX during the training period (*n* = 50)

Regarding saccade dynamics, the ANOVA for average saccade acceleration did not show a significant GROUP x TIME interaction (F_1,48_ = 2.30, *p* = 0.136, ηp2 = 0.05). Furthermore, the ANCOVA analysis did not reveal a significant GROUP difference between the stroboscopic and non-stroboscopic groups in post-training saccade acceleration (F_1,45_ = 0.68, *p* = 0.415, ηp^2^ = 0.02). Pearson product-moment correlation analyses indicated a negative relationship (*r* = -0.281, *p* = 0.048) between training-induced changes in saccade acceleration and REAC-INDEX (Fig. 4).

## Discussion

This study aimed to examine the impact of a six-week stroboscopic intervention on the visuomotor performance of young volleyball players during agility tasks. The results of this study provide compelling evidence supporting the effectiveness of stroboscopic training in improving visuomotor reaction speed, as indicated by the REAC-INDEX score. Furthermore, while the differences between groups did not reach statistical significance, there was a correlation observed between the improvements in visuomotor reaction speed performance and saccade acceleration. These findings suggest a notable influence of saccade dynamics on reactive goal-directed movements.

The enhanced visuomotor performance in the stroboscopic group supports previous research. For instance, a study on elite youth badminton players examined the effects of stroboscopic training in both lab and field settings [26]. However, when it came to specific field tasks like badminton’s smash-defense, the differences compared to the control group were not statistically significant, indicating mixed effects. In the present study, initial observations revealed significant variability in the REAC-INDEX following the intervention. Subsequent analysis using ANCOVA confirmed a significant group effect on the RA and REAC-INDEX as well. Employing ANCOVA, which is known for its superior statistical power compared to ANOVA [38], further strengthens the evidence supporting the effectiveness of stroboscopic training for volleyball players, especially in terms of reducing the RA and REAC-INDEX times.

Our study has significant practical implications, particularly in the context of team sports where reactive goal-directed movements, such as agility, play a crucial role in performance [21]. Traditionally, agility training has focused improving running speed, running technique, balance, and lower limb strength and power [18, 39–41]. However, our study reveals that the improvements in RA during the stroboscopic intervention were not solely a result of changes in running speed, as evidenced by the lack of significant differences in CODS between the experimental or control group. Considering the positive impact of stroboscopic training on RA performance, particularly in the aspect of visuomotor reaction speed, it seems that the benefits of this type of training may also extend to injury prevention. High-intensity agility movements, such as rapid changes of direction and deceleration, are known to be associated with non-contact lower limb injuries [42]. While this aspect was not explored in our article, this observation points to a promising path for future research into how stroboscopic training could potentially help reduce the risk of sports-related injuries. Previous research has indicated that decreased reaction time is moderately correlated with reduced sagittal plane knee motion and increased frontal plane knee loading among collegiate athletes with a history of sports-related concussion [11], which justifies exploring injury prevention through the application of stroboscopic training in the future.

Our findings revealed a direct correlation between changes in saccade dynamics and visuomotor reaction speed performance, as measured by the REAC-INDEX. In the analyzed relationship for the entire study sample, we observed that greater improvements in REAC-INDEX were accompanied by an increase in saccade acceleration. This suggests that the acceleration of saccades may potentially underlie the observed enhancements in visuomotor reaction speed performance during RA. Wilkins and Gray [43] explored ball-catching skill development over six weeks with stroboscopic intervention. Their study found no significant differences in catching skills or in a suite of visual tests, including visual and motion perception assessments. However, a notable correlation emerged between improved ball-catching performance and visual test scores. These observations may suggest that the influence of visual and oculomotor functions observed in our studies on task performance relates to their complexity. In simple motor tasks, intermittent visual input might suffice, augmented by feedback from other modalities like vestibular responses [44]. But in dynamic, complex movements, such as in RA tasks, oculomotor and visual functions become more crucial.

Furthermore, the utilization of stroboscopic training holds the potential to enhance visuomotor reaction function by triggering functional neural plasticity in the visual motion system [28], including saccade acceleration as a coordinated process involving the cerebral cortex, particularly the frontal eye fields, and the superior colliculus [45]. This aspect is particularly significant for volleyball training, as previous research in this sport has highlighted the importance of various oculomotor functions that significantly impact skilled player performance. Specifically, experienced volleyball players have demonstrated superior eye movement dynamics compared to non-athletes or beginners [5, 34].

The present study provides new insights into the relationship between stroboscopic training and visuomotor processes among young volleyball players. However, it is important to acknowledge the limitations of this study. Firstly, the assessment of saccade dynamics in the study was conducted using a laboratory-based test, which may have influenced the observed correlation between changes in visuomotor reaction during reactive agility and saccade acceleration. To validate this relationship, future research should concentrate on directly evaluating saccade dynamics during the execution of reactive agility tasks in real-life settings. Secondly, the inclusion of a retention test in this study was lacking, which leaves uncertainties regarding the sustained improvement of visuomotor performance and saccadic acceleration over the long term after the cessation of stroboscopic training. Thirdly, as we have not evaluated any specific far-transfer tasks, we are unable to confirm further advantages of stroboscopic intervention, such as improved game performance. Additionally, in future research, it would be valuable to consider the position on the court as a differentiating variable in visuomotor processing during reactive goal-directed movements.

## Conclusion

This study provides important insights into the trainability of sport-specific visuomotor processing using stroboscopic eyewear among young volleyball players. The use of stroboscopic training has been found to enhance visuomotor reaction speed in on-field specific skills. Furthermore, visuomotor performance gains could potentially be mediated by saccadic adaptations. Stroboscopic training shows promise as an effective method for enhancing visuomotor processing and its relevance in sport-specific training scenarios. By specifically targeting visuomotor reaction speed, coaches and athletes can incorporate stroboscopic training as a valuable addition to their training regimens, aiming to improve reactive agility and ultimately enhance on-field performance.

### Electronic supplementary material

Below is the link to the electronic supplementary material.

Supplementary Material 1

## Data Availability

The dataset supporting the conclusions of this article is included within the article (and its additional file).

## Abbreviations

CODS: change of direction speed
RA: reactive agility
REAC INDEX: visuomotor reaction speed
